# Cassava–soybean intercropping alleviates continuous cassava cropping obstacles by improving its rhizosphere microecology

**DOI:** 10.3389/fmicb.2025.1531212

**Published:** 2025-02-10

**Authors:** Huixian Chen, Lixia Ruan, Sheng Cao, Wen He, Haixia Yang, Zhenhua Liang, Hengrui Li, Wanling Wei, Zhenling Huang, Xiu Lan

**Affiliations:** ^1^Cash Crops Research Center, Guangxi South Subtropical Agricultural Science Research Institute, Longzhou, China; ^2^Cash Crops Research Institute, Guangxi Academy of Agricultural Sciences, Nanning, China

**Keywords:** cassava, soybean, intercropping, continuous cropping obstacles, metabolite, soil metabolome

## Abstract

**Introduction:**

Continuous cropping is the main cause of cassava yield reduction. To find an effective method to alleviate the obstacle of cassava continuous cropping and explore the effect of cassava–soybean intercropping, this study analysed the differences in cassava agronomic traits, yield, soil physicochemical properties, microbial community structure, and metabolites between cassava single cropping (M) and cassava–soybean intercropping (MD) and its effects on continuous cassava cropping soil.

**Methods:**

The correlations between yield, agronomic traits, soil physicochemical properties, microbial diversity, and metabolites were explored, and the effect of the cassava–soybean intercropping model on cassava soil was revealed.

**Results:**

The results showed that compared with group M, soil pH, porosity, organic matter, available nitrogen, and fresh potato yield in the MD group significantly increased by 8.59, 13.66, 20.68, 23.29, and 50.61%, respectively, and soil bulk density significantly decreased by 9.68%. Soil bacterial community diversity in the MD group did not change significantly but had significant effects on soil fungal community diversity. The relative abundances of *Trichoderma* and *Micropsalliota* in the MD group were significantly upregulated. The contents of phenol glucuronide, 2,3-butanediol, L-phenylalanine, deoxyguanosine, other carbohydrates, alcohols, purine nucleotides, and amino acids in the soil of the MD group were significantly upregulated. Organic acids, such as fumaric acid, succinic acid, phosphoenolpyruvic acid, decreased significantly. Correlation analysis showed that *Trichoderma* was significantly negatively correlated with fumaric acid, succinic acid, phosphoenolpyruvic acid, and soil bulk density. However, there was significant positive correlation with phenol glucuronide, alpha-CEHC deoxyguanosine and other carbohydrates, nucleotide substances, organic matter, and pH. Phenol glucuronide, 2,3-butanediol, L-phenylalanine, deoxyguanosine and other carbohydrates, alcohols, purine nucleotides, and amino acids were significantly positively correlated with organic matter, available nitrogen, soil porosity, and pH.

**Discussion:**

Therefore, cassava–soybean intercropping can effectively alleviate the obstacles of continuous cassava cropping by affecting the accumulation of metabolites and microbial community structure in continuous cropping soil, thereby improving the adverse factors of severe soil acidification, soil compaction, and nutrient decline.

## 1 Introduction

Cassava (*Manihot esculenta* Crantz) is a plant in the Euphorbiaceae family, belonging to the *Manihot* genus. It is one of the three major potato crops in the world, known as the “king of starch,” and is an important food source in tropical and subtropical regions globally (Li et al., 2017). Cassava has important industrial uses in the production of a wide range of products, such as starch, ethanol, feed, pharmaceuticals, cosmetics, and biopolymer (Ceballos et al., 2004; Uchechukwu-Agua et al., 2015). Under the influence of land resources, climatic conditions, planting habits, and economic benefits, continuous cropping of cassava is widespread. However, this causes soil compaction, acidification, nutrient loss, and microbial community structure imbalance, resulting in growth restriction and has become the main reason for cassava yield reduction (Adjei et al., 2017; Zhou, 2017; Liu, 2020; Liang et al., 2017). Currently, there are few studies on the mitigation measures for continuous cassava cropping, and it is difficult to develop effective measures. Therefore, finding ways to alleviate the limitations to continuous cassava cropping has become an urgent and important research topic.

Intercropping is the practice of planting two or more crops at the same time on the same piece of land and is widely regarded as an effective method for maintaining and improving soil quality and increasing crop yield (Mao et al., 2015; Chen et al., 2021; Cuartero et al., 2022). Intercropping refers to the coexistence of multiple crops in a soil system. The root systems of the crops interact with each other, thus exerting a positive impact on the microorganisms and nutrients of the symbiotic soil (Lian et al., 2019; Zhang et al., 2021), such as improving soil available nitrogen, phosphorus, and potassium; organic matter content; catalase activity; bacterial community diversity; and the abundance of beneficial microorganisms, thus promoting crop growth (Zhou et al., 2011; Tang et al., 2020; Zhao et al., 2022; He et al., 2023; Zheng et al., 2023). In addition, root secretions can provide a carbon source for bacteria and can also be used as signalling substances to affect the interaction between different crops, thus improving the availability of soil nutrients (Lian et al., 2002; Zhang et al., 2016; Jiang et al., 2022; Liu et al., 2022). In view of the many advantages of intercropping for soil improvement, it is also widely used in production practices to alleviate the limitations of continuous cropping. Researchers have found that intercropping can optimise the composition of beneficial bacteria in continuous cropping soil, regulate soil microecology, and reduce the autotoxicity of allelopathic substances (Gao et al., 2021; Zeng et al., 2020); however, the mechanisms of alleviating continuous cropping barriers differ among different intercropping combinations.

Soybean can be symbiotic with rhizobia to fix nitrogen, improve soil nitrogen content, and recruit beneficial microorganisms from soil according to the growth needs of plants (Mendes et al., 2014; Gao et al., 2021). It is considered a “pioneer crop” for degraded soil improvement (Canfield et al., 2010; Cheng et al., 2023), For example, sugarcane–soybean intercropping reduces soil pH and increases soil organic carbon, dissolved organic carbon, and available nitrogen (Zhang et al., 2023). The maize–soybean intercropping model can increase soil organic matter and total nitrogen content, improve soil fertility and the carbon to nitrogen ratio, change the rhizosphere bacterial community, increase the diversity of microbial communities, and increase the abundance of beneficial microorganisms in the soil rhizosphere (Chang et al., 2022; Te et al., 2022). Cassava–soybean intercropping is a highly efficient intercropping model that has garnered considerable attention (Ennih et al., 2006; Wei et al., 2015; Harsono, 2017); however, the effects of this model on soil microorganisms and root exudates remain unknown, especially in continuous cassava cropping soil. Therefore, this study analysed the differences in and explored the correlation between cassava agronomic traits, yield, soil physicochemical properties, soil nutrients, rhizosphere microbial community structure, and metabolites in continuous cassava cropping and cassava–soybean intercropping. The effects of the cassava–soybean intercropping model on continuous cassava cropping soil provide a theoretical basis for improving continuous cassava cropping soil.

## 2 Materials and methods

### 2.1 Field experiment materials

The cassava variety used in this experiment was ‘Xinxuan 048’ bred by Guangxi University, and the soybean variety used was ‘Guichundou8’ bred by the Institute of Cash Crops, Guangxi Academy of Agricultural Sciences.

### 2.2 Field experimental design

The experiment was conducted at the Guangxi Institute of South Tropical Agricultural Science, Longzhou County, Chongzuo City, Guangxi from 2021 to 2023. The climate of the test site was subtropical monsoon, the soil was red loam, the planting system was cassava for one season a year, and the planting period of cassava was more than 5 years. Before the experiment, the soil pH at 0–20 cm depth was 4.80, total nitrogen was 1.03 g/kg, available nitrogen was 95.4 mg/kg, organic matter was 25.40 g/kg, available phosphorus was 16.83 mg/kg, and available potassium was 186.0 mg/kg.

**Figure 1:**
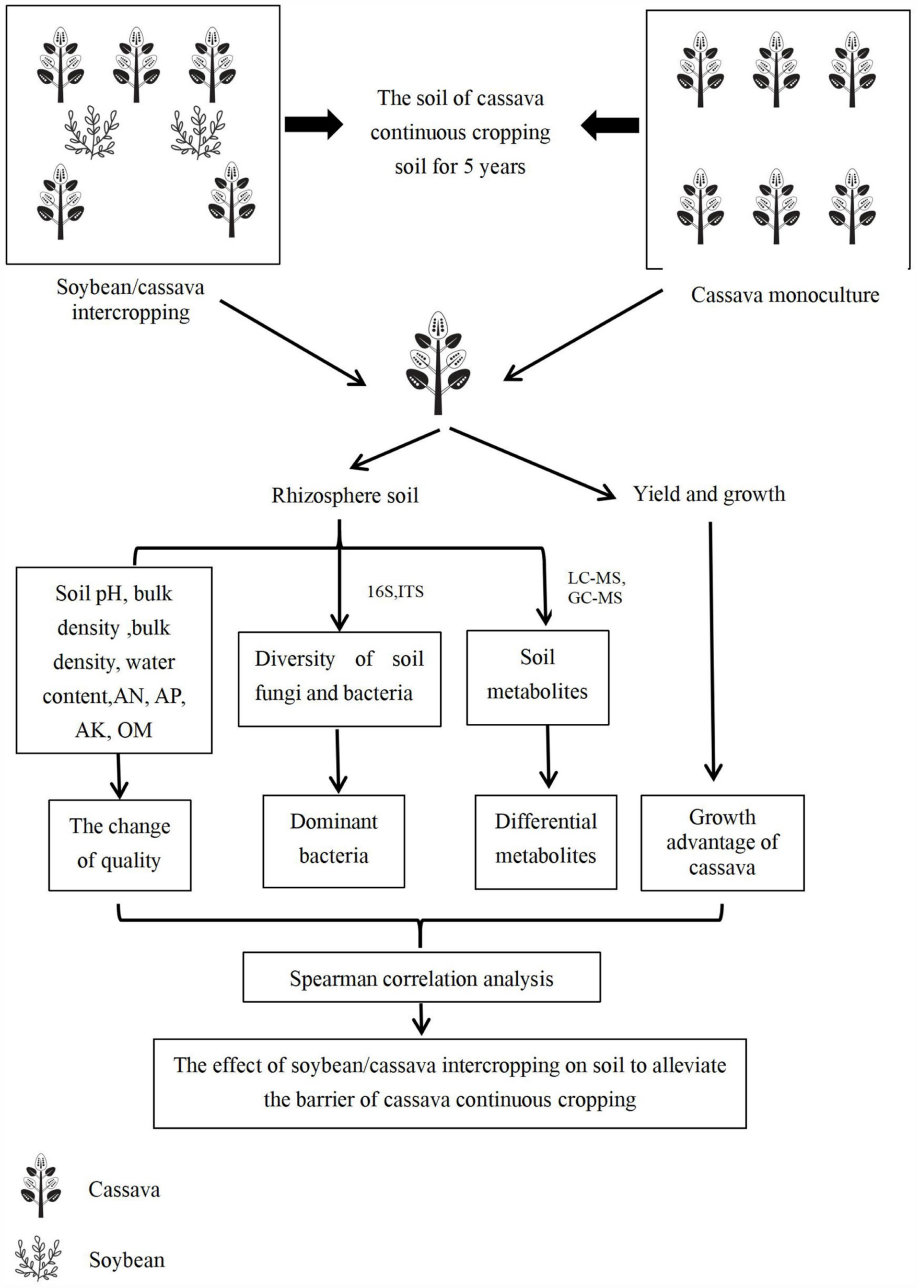
The diagrammatic overview of the experimental workflow.

The test material was planted on 15 March, soybeans were harvested in July, and cassava was harvested on December 15. A single factor experimental design was implemented, with the planting mode as the independent variable. In cassava continuous cropping soil, the experimental treatments were divided into M and MD. Cassava was planted in a single row, and soybeans were planted on both sides of the cassava. The experiment was repeated six times, and the plots were arranged randomly. Based on the characteristics of cassava growth requiring fertiliser and the conventional high-yield cultivation method of cassava, the type and amount of fertilisation in this experiment were determined. Compound (N: P_2_O_5_:K_2_O = 15:10:10) and organic (7,500 kg/ha) fertilisers were applied as base fertilisers in each experiment plot, and no other fertilisers were applied during the growth of cassava and soybean. The row spacing of cassava was 80 × 100 cm, and the row spacing of soybean was 10 × 60 cm. The plant height, stem diameter, cassava yield, soil porosity, bulk density, and water content were determined during the cassava and soybean symbiosis period (June). Rhizosphere soil was collected to determine the content of soil available nitrogen (AN), available potassium (AK), available phosphorus (AP), organic matter (OM), soil pH, microbial diversity, and metabolites. The workflow chart shows as Figure 1.

### 2.3 Sample collection and preparation

The rhizosphere soil was collected within 2 mm from the root, and soil samples were collected using the “five-point sampling method.” Five sampling points were selected in each plot, that is, five cassava plants. Plant residues on the soil surface were removed during sampling, and cassava was removed. The rhizosphere soil was collected using the shake-off method. The soil with strong adhesion to the root surface was removed, and retained as the rhizosphere soil for the experiment. The soil samples were placed in sterile sealed bags and stored in iceboxes. After returning to the laboratory, the soil samples from five sample points in each plot were mixed. Each of the soil samples was sieved(<2 mm) and then divided into two aliquots in sealed bags. One soil sample was quickly frozen with liquid nitrogen and stored in a refrigerator at −80°C to determine microbial diversity and metabolites. Another soil sample was air-dried and screened through a 100 mesh to determine the AN, AK, AP, OM, and pH.

### 2.4 Determination of agronomic traits and cassava yield

Ten consistently growing plants were collected continuously from each plot; the height, stem diameter, and fresh root weight of each plant were measured; and the average was calculated. The height of the plant was measured with a tape measure from the ground to the top of the plant, and the diameter of the stem was measured using a Vernier calliper to determine the diameter of the stem 20 cm from the ground. The length of the root tuber was measured with a straight ruler to determine the its length to the tail. The diameter of the root tuber was measured in the middle of the root, and the fresh potato was weighed with a scale to determine the weight of the whole cassava root.

### 2.5 Determination of physicochemical properties of cassava rhizosphere soil

Physicochemical properties of the rhizosphere soil were measured, namely soil porosity, bulk density, water content, AN, AK, AP, OM, and pH.

The ring knife method was used to determine the soil bulk density: the soil was extracted with a ring knife and dried. The soil bulk density referred to the mass of the dried soil/the volume of the ring knife. Soil moisture (%) = (original soil weight − dried soil weight)/dried soil weight ×100%, and the specific gravity of soil was determined using the gravity bottle method. Soil porosity (%) = (1 − bulk weight/specific gravity) × 100.

According to previously reported methods (Zhang et al., 2018), AN was measured by alkaline hydrolysis diffusion method. For soil AP and AK, the soils were first extracted with 0.5 mol/L sodium bicarbonate solution and 1 mol/L ammonium acetate solution, respectively, and then measured by Continuous Segmented Flow Analyzer (AutoAnalyzer 3, Seal Analytical, Germany). The organic matter was determined by potassium dichromate oxidation method. Soil pH value was measured with a pH meter. 10 g of soil sample air-dried through a 1 mm screen was placed in a 25 mL beaker, 10 mL of distilled water was added and mixed, and the pH value of the suspension was determined with a corrected pH meter.

### 2.6 Microbial analysis

Total genomic DNA was extracted using a MagPure Soil DNA LQ Kit (Magan) following the manufacturer’s instructions. DNA concentration and integrity were measured using a NanoDrop 2000 (Thermo Fisher Scientific, USA) and agarose gel electrophoresis. Extracted DNA was stored at −20°C until further processing. The extracted DNA was used as a template for the PCR amplification of the 16S rRNA/ITS and ITS rDNA genes using barcoded primers and Takara Ex Taq (Takara). The bacterial 16S rRNA gene V3-V4 high variant region was amplified using the universal primers 343F (5′-TACGGRAGGCAGCAG-3′) and 798R (5′-AGGGTATCTAATCCT-3′) (Nossa et al., 2010). For fungal diversity analysis, the ITS variable regions were amplified using universal primers ITS1 (5′-CTTGGTCATTTAGAGGAAGTAA-3′) and ITS2 (5′GCTGCGTTCTTCATCGATGC-3′). Amplicon quality was visualised using agarose gel electrophoresis. PCR products were purified using AMPure XP beads (Agencourt) and amplified in another round of PCR. After purification with AMPure XP beads, the final amplicon was quantified using a Qubit dsDNA Assay Kit (Thermo Fisher Scientific, USA). The concentrations were adjusted for sequencing, which was performed on an Illumina NovaSeq 6000 with 250 bp paired-end reads (Illumina Inc., San Diego, CA; OE Biotech Company; Shanghai, China).

### 2.7 Metabolomics analysis

#### 2.7.1 Liquid chromatography/mass spectrometry non-targeted metabolomics

First, 500 mg of the soil sample was accurately weighed into a 1.50 mL EP tube with an internal standard (2 μg/mL), and 1 mL of methanol–water (V:V = 1:1) was added. Then, two small steel beads were added, precooled in a − 20°C freezer for 2 min, and the sample was ground at 60 Hz for 2 min. Subsequently, the samples were subjected to ultrasonic extraction in an ice-water bath for 10 min. Next, the sample was allowed to stand at −20°C for 2 h. Finally, the sample was centrifuged for 10 min (12,000 rpm, 4°C). Then, 150 μL of the supernatant was aspirated with a syringe, and the organic phase was filtered through a 0.22 μm pinhole filter, transferred to an LC injection vial, and stored at −80°C until LC–MS analysis was performed.

#### 2.7.2 Gas chromatography/mass spectrometry non-targeted metabolomicsgas

Similar to the above steps, first, 30 mg of hippocampal tissue was accurately weighed into a 1.50 mL EP tube with an internal standard (20 μL), and, 600 μL of methanol–water (V:V = 4:1) was added. Then, two small steel beads were added, and after precooling in a −20°C freezer for 2 min, the sample was ground at 60 Hz for 2 min. Next, 120 μL chloroform was added, and the sample was rotated for 2 min and then ultrasonically extracted in an ice-water bath for 10 min. The sample was allowed to stand at −20°C for 30 min then centrifuged at 4°C at 13,000 rpm for 10 min. Then, 150 μL of the supernatant was transferred into a standard glass flask. Next, the sample was dried with a centrifugal concentrator dryer, and 80 μL of methoxylamine hydrochloride pyridine solution (15 mg/mL) was added to the standard glass vials. The vials were shaken for 2 min and then shaken in an incubator at 37°C for 90 min for the oxime reaction. After the samples were removed, 50 μL of bis (trimethylsilyl)trifluoroacetamide (BSTFA) (containing 1% chlorotrimethylsilane) derivatizing reagent and 20 μL of n-hexane were added, 10 types of internal standards (10 μL) were added, and the samples were vortexed for 2 min and reacted at 70°C for 60 min. After removing the heat block, the samples were incubated at room temperature for 30 min before GC–MS analysis. LC–MS and GC–MS analyses were performed by OE Biotech Co., Ltd. (Shanghai, China).

### 2.8 Statistical analysis

Statistical analysis was performed using IBM SPSS Statistics. Multiple comparisons among experiments were performed by one-way analysis of variance (ANOVA) with the least significant difference (LSD) test for multiple comparisons, and *p* < 0.05 was considered to indicate statistical significance. In the 16S rRNA and ITS rDNA analysis, the ASVs were subjected to alpha diversity and beta diversity analysis using QIIME2 software, and the microbial diversity in the samples was estimated using alpha diversity that included the Chao1, Shannon, ACE, and Simpson indices; the unweighted Unifrac distance matrix performed by the R package was used for unweighted Unifrac Principal Component Analysis (PCA) to estimate beta diversity. The R package was used to analyse significant differences between groups using ANOVA. The linear discriminant analysis effect size (LEfSe) method was used to compare the taxonomic abundance spectrum. Orthogonal partial least squares discriminant analysis (OPLS-DA) was used to screen differentially abundant metabolites, 7-fold cross validation and 200 response permutation tests (RPT) were used to evaluate the quality of the model, and Variable Importance of Projection (VIP) values obtained from the OPLS-DA model were used to rank the overall contribution of each variable to group discrimination. Two-tailed Student’s t-test was used to verify whether the metabolite differences between the groups were significant. Differential metabolites were selected with VIP values >1.0 and *p*-values <0.05. Differential metabolites were used for KEGG pathway enrichment analysis (http://www. Genome.jp/kegg/), and association analysis was performed using Spearman correlation analysis method.

## 3 Results

### 3.1 Soil physicochemical properties and cassava growth under two planting modes

The soil pH, porosity, water content, OM, AK, AP, and AN in the MD group increased by 8.59, 13.66, 7.78, 20.68, 4.26, 5.50, and 23.29%, respectively, compared with group M. Soil bulk density decreased by 9.68%, and soil pH, porosity, bulk density, OM, and AN differed significantly between the two planting modes (Table 1). Compared with group M, the plant height, stem diameter, and yield per plant of cassava in the MD group increased by 14.10, 14.99, and 50.61%, respectively, and the yield per plant of cassava in the two planting modes was significantly different (Table 2). Therefore, the cassava–soybean intercropping model can alleviate adverse factors, such as soil compaction, nutrient decline, and soil acidification in continuous cassava cropping, thus promoting cassava growth.

**Table 1:** Differences in physicochemical properties of cassava rhizosphere soil for two planting modes.

Cropping pattern	pH	Porosity (%)	Bulk density (g/cm^3^)	Water content (%)	Organic matter (g/kg)	Available potassium (mg/kg)	Available phosphorus (mg/kg)	Available nitrogen (mg/kg)
M	4.54 ± 0.18b	33.78 ± 0.50b	1.63 ± 0.11a	18.89 ± 0.40a	19.54 ± 1.23b	156.35 ± 10.97a	49.05 ± 5.29a	95.57 ± 4.16b
MD	4.93 ± 0.13a	37.05 ± 2.35a	1.41 ± 0.14b	20.36 ± 1.04a	23.58 ± 1.83a	163.01 ± 10.45a	51.75 ± 1.89a	117.83 ± 9.22a

Values in the table are mean ± standard deviation, and different lowercase letters in the same column indicate significant differences between treatments (*p* < 0.05).

**Table 2:** Differences in cassava growth for two planting modes.

Cropping pattern	Plant height	Stem diameter	Yield (kg/ per plant)
M	1.56 ± 0.12a	30.22 ± 4.44a	1.64 ± 0.39b
MD	1.78 ± 0.23a	34.75 ± 9.25a	2.47 ± 1.10a

Values in the table are mean ± standard deviation, and different lowercase letters in the same column indicate significant differences between treatments (*p* < 0.05).

### 3.2 Characteristics of soil microbial community structure under the two planting modes

#### 3.2.1 Differences in soil microbial species diversity under the two planting modes

**Figure 2:**
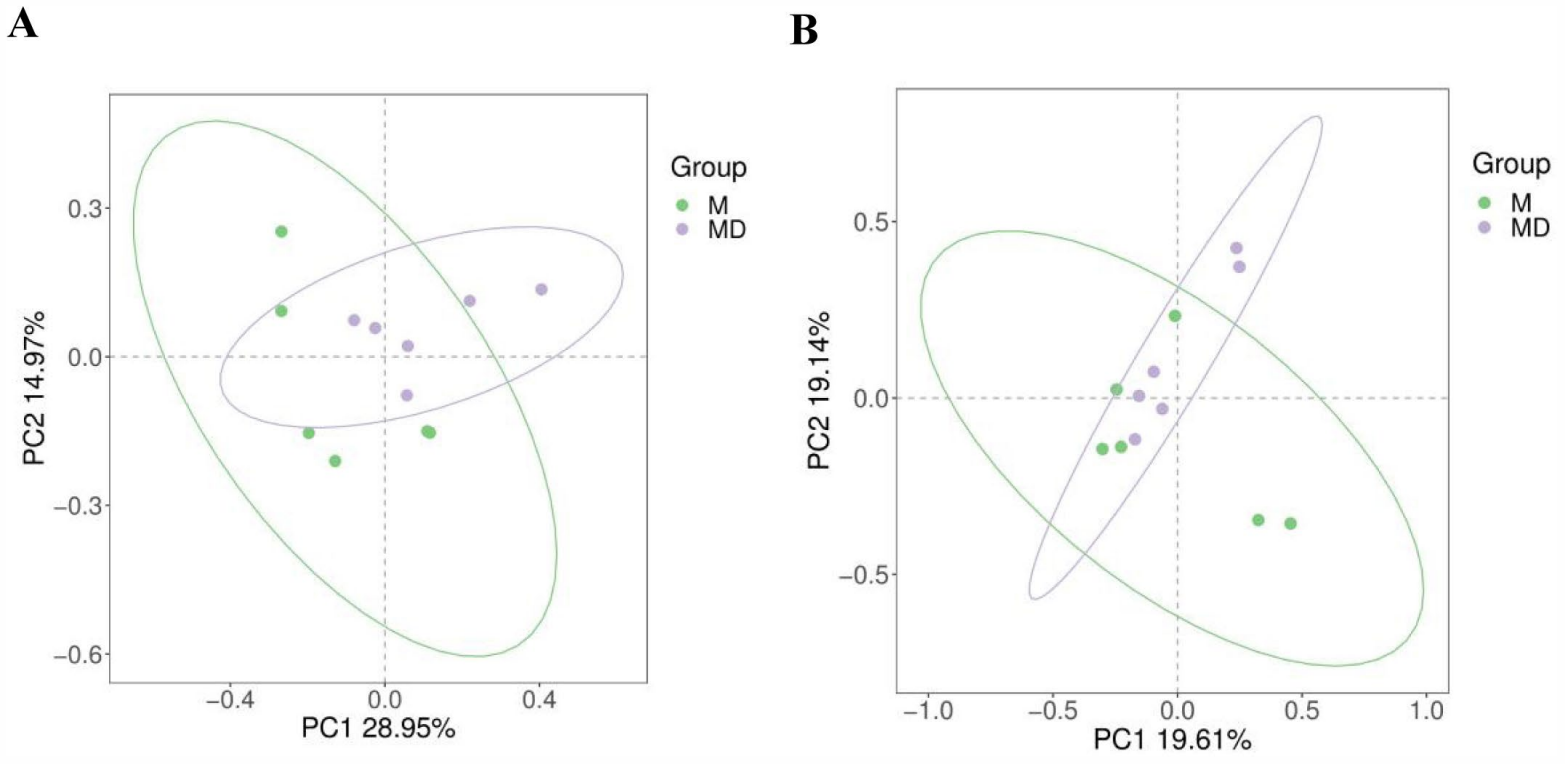
Principal Component Analysis (PCA) of microbial communities in cassava rhizosphere soils for two planting modes. **(A)** Bacteria; **(B)** Fungi.

**Figure 3:**
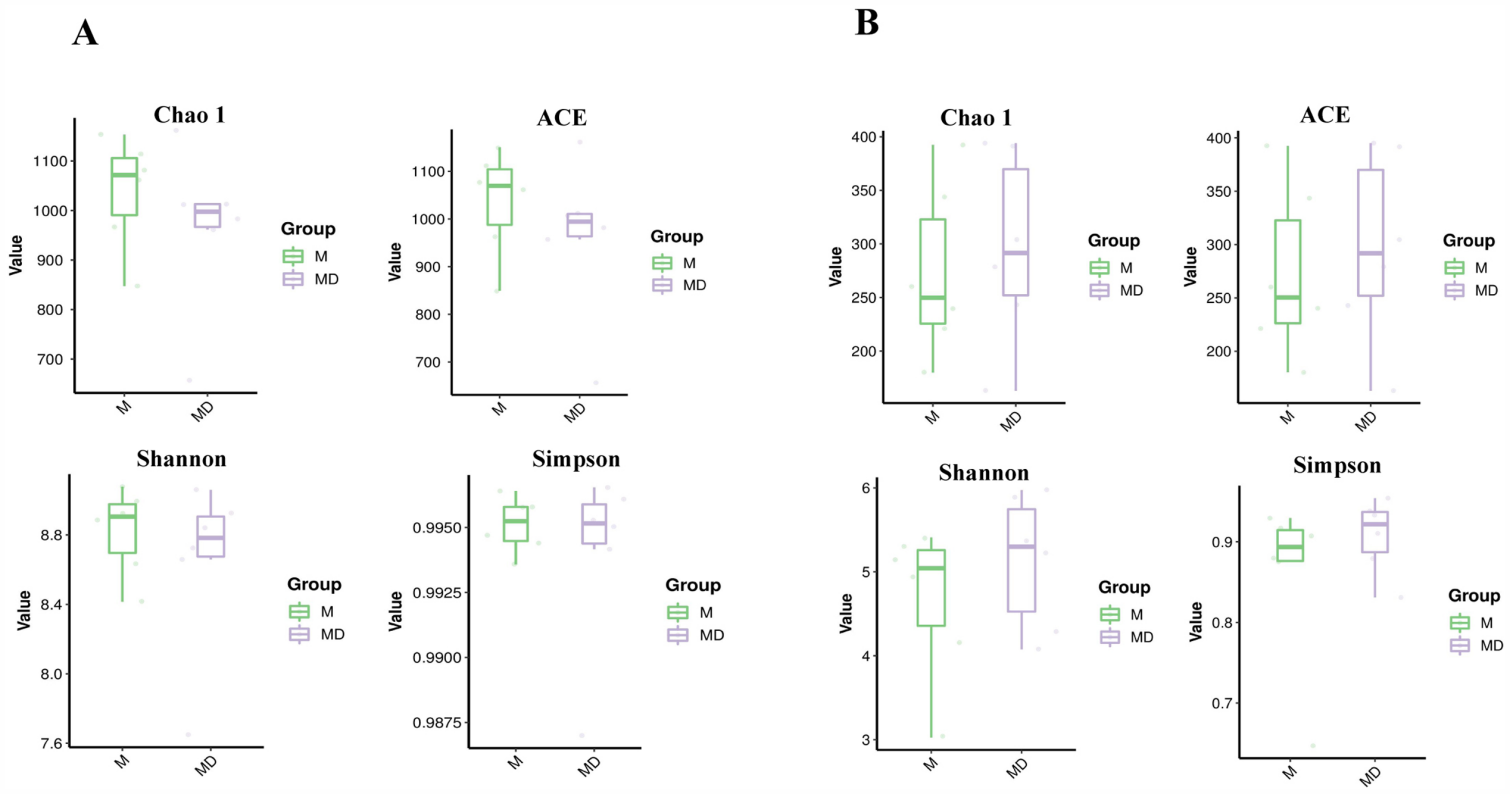
Analysis of Chao 1 index, ACE index, Shannon index and Simpson index related to alpha diversity in cassava rhizosphere soils for two planting modes. **(A)** Bacteria; **(B)** Fungi.

The PCA results of microbial species diversity showed that compared to the M group, only insignificant subtle changes were observed in the MD group (Figure 2). The box plot of the alpha diversity index revealed that compared with group M, the Chao1, Shannon, ACE, and Simpson indices of bacteria in the MD group decreased, whereas those of fungi increased. Although there was no significant difference in the alpha diversity index of bacteria and fungi between the two planting patterns, the bacterial diversity and richness of soil in the MD group showed a downward trend, whereas the fungal diversity and richness showed an increasing trend, indicating that cassava–soybean intercropping had the potential to improve soil fungal diversity (Figure 3).

#### 3.2.2 Difference in soil dominant microbial community composition and relative abundance under two planting modes

**Figure 4:**
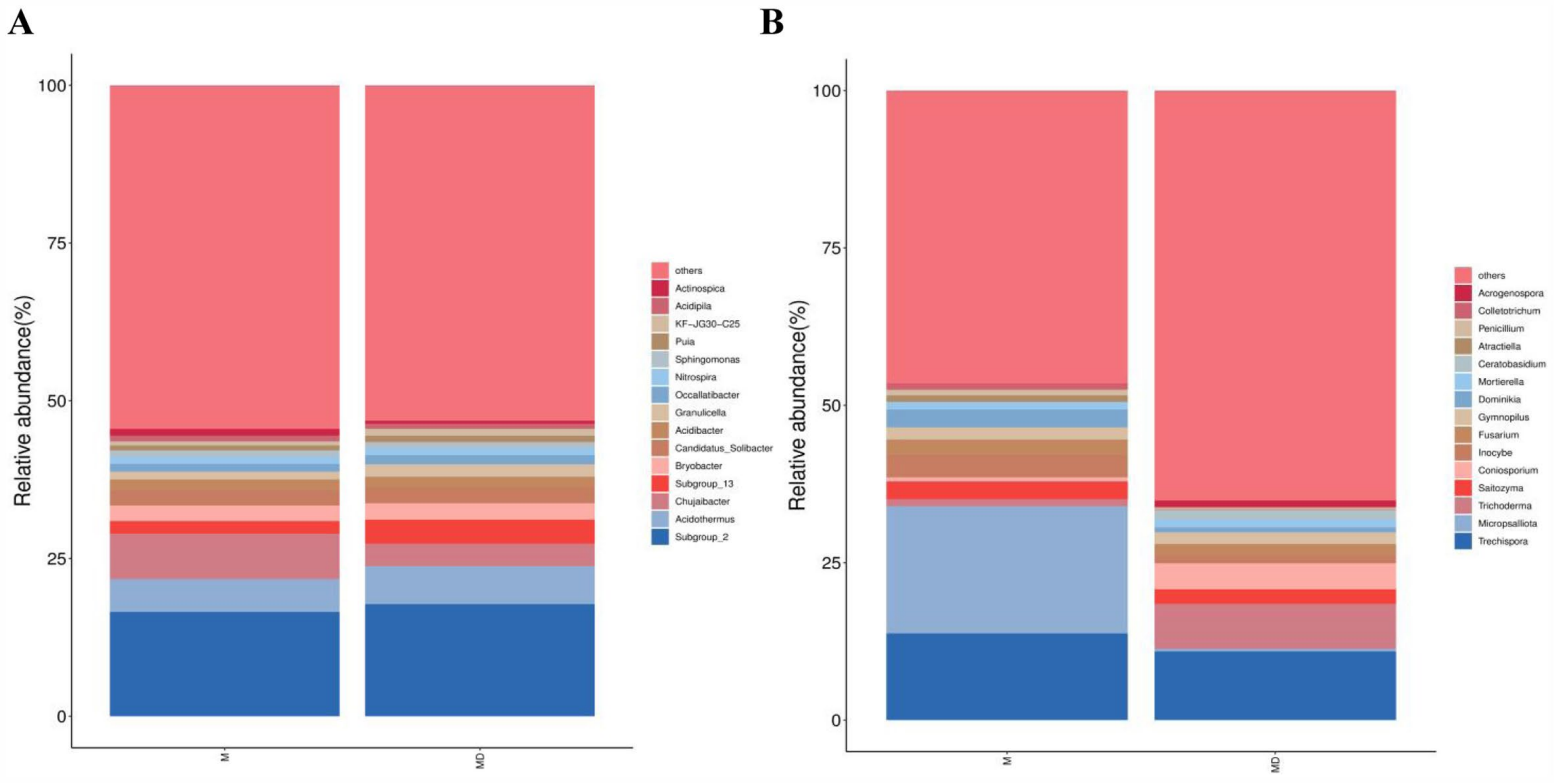
Relative abundance of the top 15 microbial communities at the genus level in cassava rhizosphere soils for two planting modes. **(A)** Bacteria; **(B)** Fungi.

**Figure 5:**
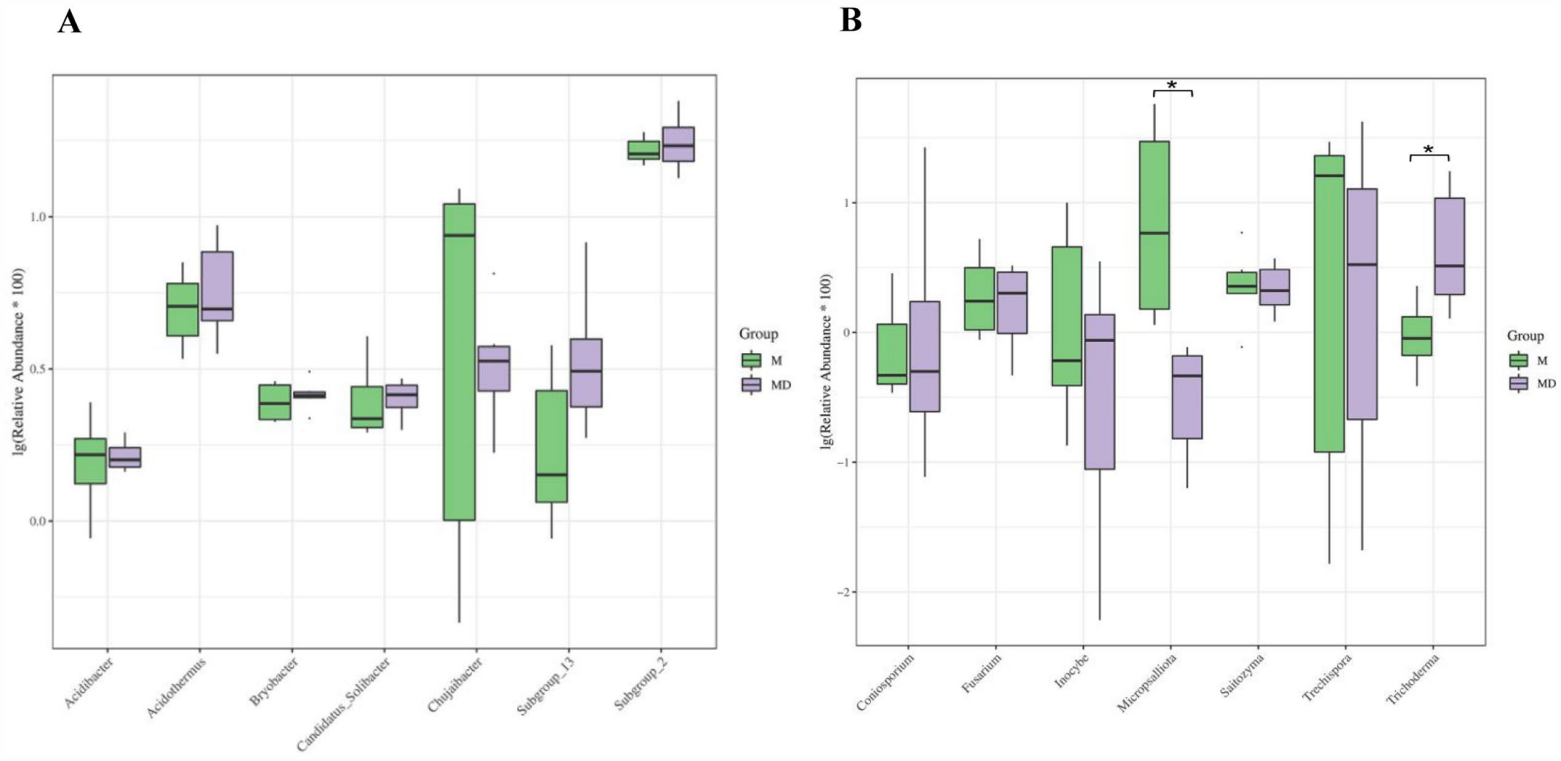
Differential analysis of dominate microbial communities composition at the genus level in cassava rhizosphere soils for two planting modes. **(A)** Bacteria; **(B)** Fungi. Six biological replicates were used in this experiment, and same letters represents not significantly different.

The top 15 most abundant bacterial and fungal communities were characterised under the two planting patterns, and microbial communities with a relative abundance >1% were identified as the dominant microorganisms. Bacterial communities with a relative abundance >1% were composed of seven genera, namely *Subgroup_2*, *Acidothermus*, *Chujaibacter*, *Subgroup_13*, *Bryobacter, Candidatus_Solibacter,* and *Acidibacter* (Figure 4A). The MD mode increased the relative abundances of *Acidothermus, Subgroup_13, Candidatus_Solibacte, and Subgroup_2* and decreased the relative abundances of *Chujaibacter, Bryobacter, and Acidibacter*. However, none of these values were significant (Figure 5A). The fungal community with a relative abundance of >1% was composed of seven genera: *Trechispora, Micropsalliota, Trichoderma, Saitozyma, Coniosporium, Inocybe, and Fusarium* (Figure 4B). The MD mode increased the relative abundances of *Trichoderma and Coniosporium* and decreased the relative abundances of *Trechispora, Micropsalliota, Saitozyma, Inocybe, and Fusarium*. The relative abundances of *Trichoderma* and *Micropsalliota* differed significantly between the two groups (Figure 5B). These results indicate that cassava–soybean intercropping had significant effects on the relative abundance of the dominant fungal community in the rhizosphere soil of cassava but had little effect on the composition and relative abundance of the dominant bacterial community in the soil.

**Figure 6:**
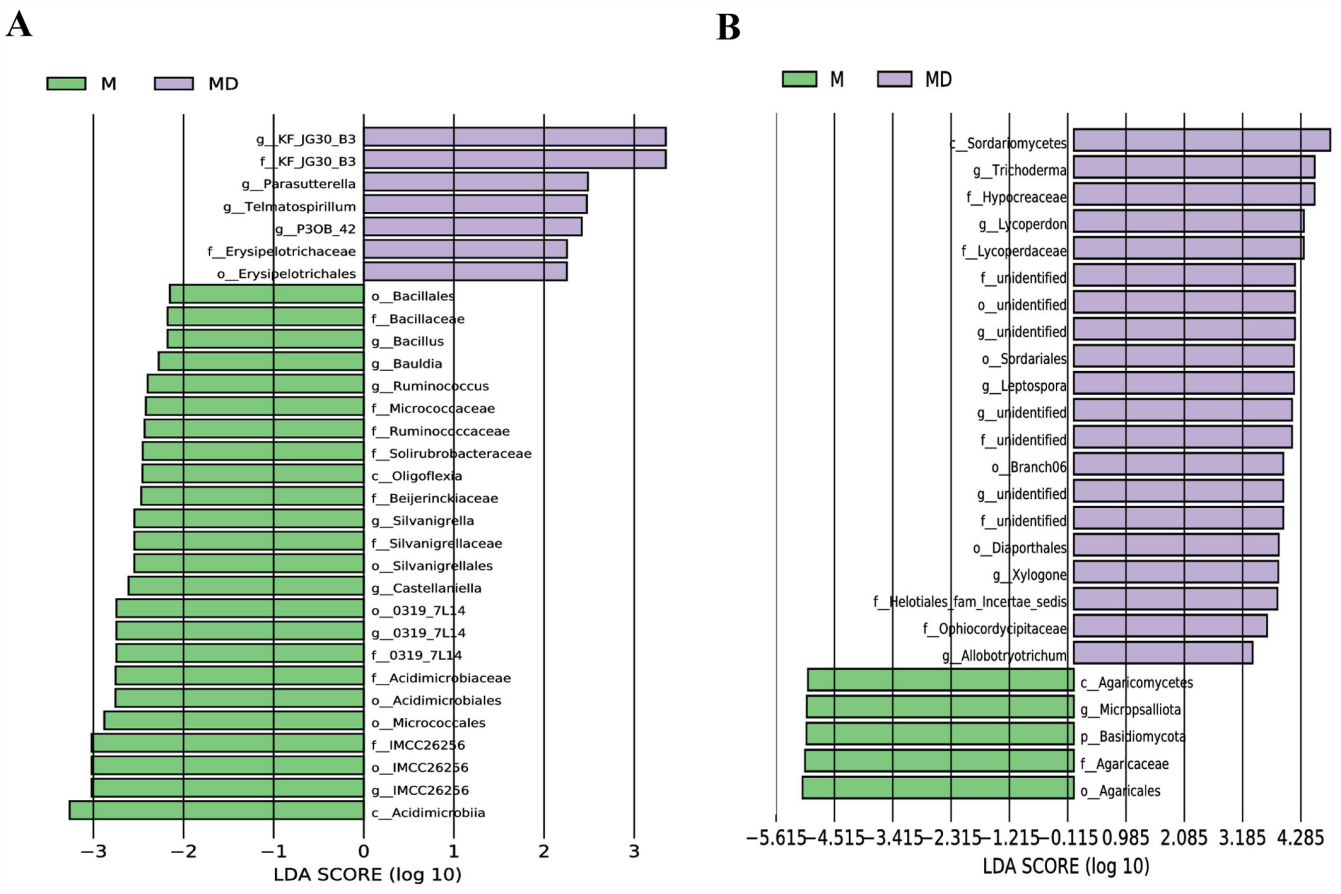
LDA Effects Size (LEfSe) analysis of microbial communities in cassava rhizosphere soils for two planting modes. **(A)** Bacteria; **(B)** Fungi.

LEfSe was used to explore the biological markers and contribution sizes of bacteria and fungi with an LDA score > 3.5 as biomarkers. LEfSe revealed that the number of labelled bacteria was lower than that of labelled fungi in both MD and M modes, and the number of different biomarkers in the MD mode was significantly higher than that in the M mode. p_Basidiomycota, *g_Micropsalliota*, and c_Agaricomycetes were marker fungi in the M mode. Nineteen phyla or genera, including c_Sordariomycetes, *g_Trichoderma*, and f_Hypocreaceae, were markers in the MD mode (Figures 6A,B). Combined with the results of microbial relative abundance difference analysis and LEfSe, the cassava–soybean intercropping model had little effect on the composition and relative abundance of the dominant bacterial community in cassava continuous cropping soil but had a significant effect on the relative abundance of the dominant fungal community in the continuous cropping soil. In particular, it affected the relative abundance of *Trichoderma and Micropsalliota*.

### 3.3 Analysis of soil metabolites in the rhizosphere under two planting modes

#### 3.3.1 Rhizosphere soil metabolites

**Figure 7:**
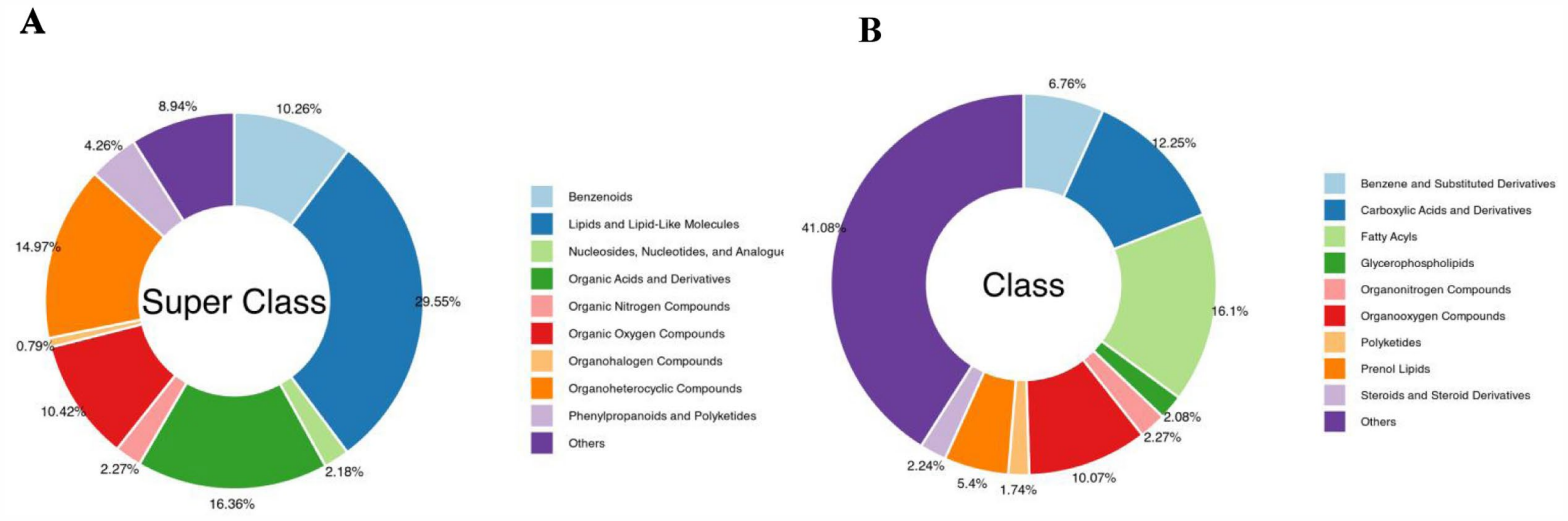
Classification of the 3,167 identified metabolites in the cassava rhizosphere soils for two planting modes. **(A)** Super Class; **(B)** Class.

The changes in soil metabolites in the cassava rhizosphere under the two planting modes were analysed using LC–MS/MS and GC–MS/MS metabolomics. There were 3,167 different metabolites detected, which were divided into nine categories: lipids and lipid-like molecules (29.55%), organic acids and derivatives (16.36%), organoheterocyclic compounds (14.97%), organic oxygen compounds (10.42%), and benzenoids (10.26%) were the main metabolites (Figure 7A). In the secondary category, fatty acids (16.1%), carboxylic acids and derivatives (12.25%), organo-oxygen compounds (10.07%), and benzene and substituted derivatives (6.76%) were the main metabolites (Figure 7B).

#### 3.3.2 Screening of different metabolites in the rhizosphere soil

**Figure 8:**
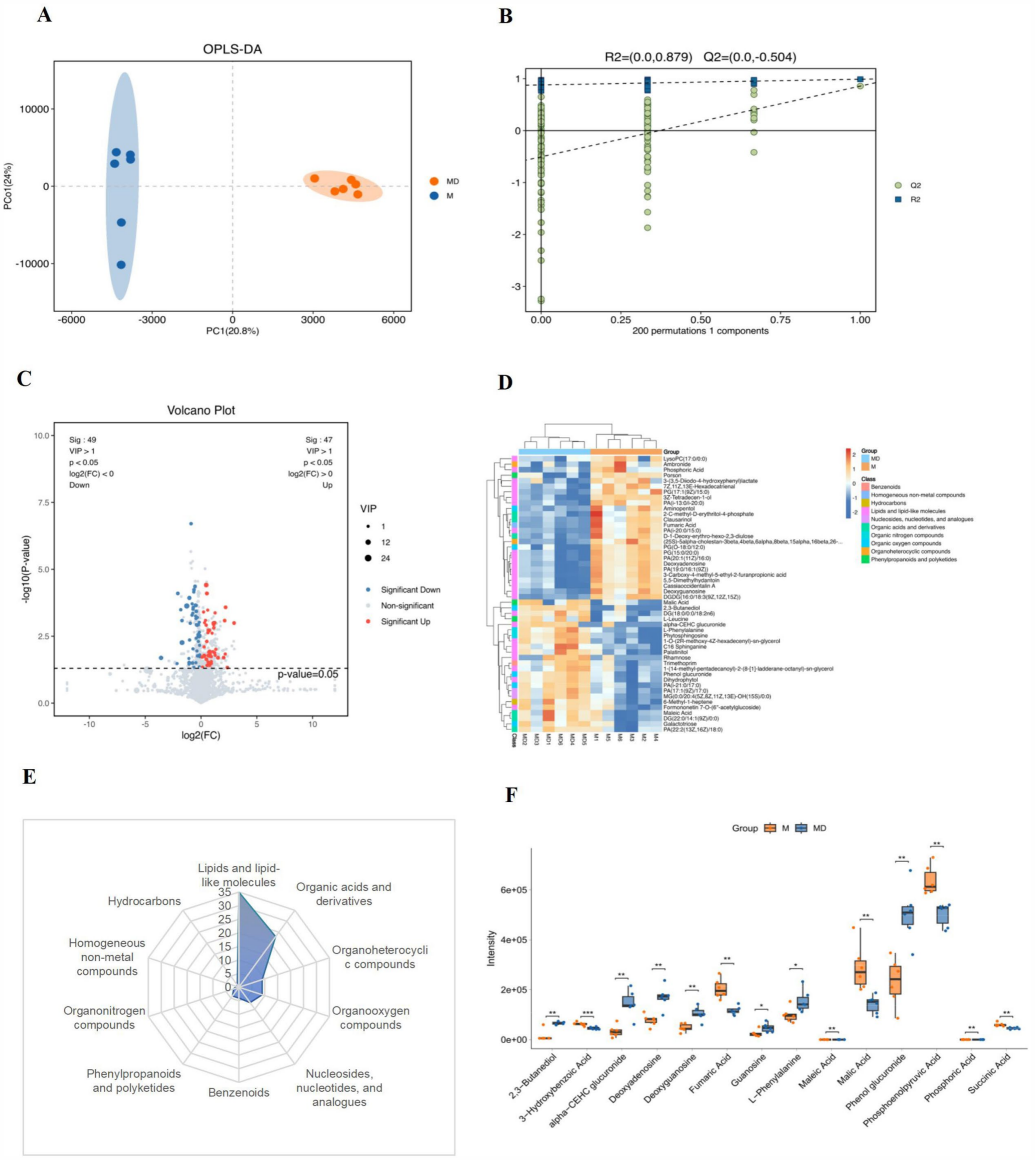
Analysis of soil metabolites in the cassava rhizosphere soils for two planting modes. **(A)** OPLS-DA; **(B)** response permutation testing (RPT); **(C)** volcano plots; **(D)** heatmap (top 50); **(E)** classification of soil DEMs; **(F)** differential analysis of soil DMEs. Six biological replicates were used in this experiment, and same letters represents not significantlydifferent.

OPLS-DA was used to analyse the changes in metabolites in the cassava rhizosphere soil. Six bioreplicates in each group clustered together, indicating the reliability of the detection data and strong homogeneity between the bioreplicates. Samples treated under MD and M were located on both positive and negative sides of PC1, indicating significant differences in the metabolites between the two groups (Figure 8A). To prevent overfitting, 7-fold cross validation and 200-times response permutation testing (RPT) were used to investigate the quality of the model. R2Y and Q2 were smaller than the R2Y and Q2 of the original model; that is, all points on the left side of the figure (substitution test) were lower than the point at the abscess coordinate of 1 (the original model), and the intercept between the regression line and the vertical axis was <0 (Figure 8),. This indicated that this model is meaningful and can be used for the subsequent screening of differential metabolites (Figure 8B).

Based on the OPLS-DA model, the variable weight value VIP > 1 and t-test *p* < 0.05 were used to screen differential metabolites. Volcanic maps showed that 98 different metabolites were screened in the two groups of samples, 49 of which were upregulated and 49 were downregulated (Figure 8C). In the heat map of the cluster analysis, the bands of the different treatments showed obvious differences, and the three metabolite bands of the same treatment showed high consistency (Figure 8D). We classified and counted the two groups of DEMs and found that they mainly included 10 classes (Level 1): lipids and lipid-like molecules (35.71%), organic acids and derivatives (23.47%), organoheterocyclic compounds (9.18%), organoheterocyclic compounds (9.18%), nucleosides, nucleotides and analogues (7.14%), benzenoids (4.08%), phenylpropanoids andpolyketides (4.08%), organonitrogen compounds (2.04%), homogeneous non-metal compounds (1.02%), and hydrocarbons (1.02%) (Figure 8E). Among these 10 types of different metabolites, the contents of carbohydrates, alcohols, amino acids, purine nucleosides, and other metabolites, such as phenol glucuronide, alpha-CEHC glucuronide, 2,3-butaneiol, L-phenyalanine, deoxyguanosine, guanosine, and deoxyadenosine, were significantly increased in the MD mode. The content of organic acids, such as fumaric acid, succinic acid, phosphonolpyruvic acid, malic acid, phosphoric acid, maleic acid, and 3-Hydroxybenzoic acid, significantly decreased in the MD mode (Figure 8F). The results showed that the metabolic processes of soil and microorganisms underwent substantial changes after soybean intercropping, mainly by changing the content of metabolites, such as carbohydrates, alcohols, purine nucleosides, amino acids, and organic acids.

### 3.4 Enrichment analysis of differential metabolic pathways

**Figure 9:**
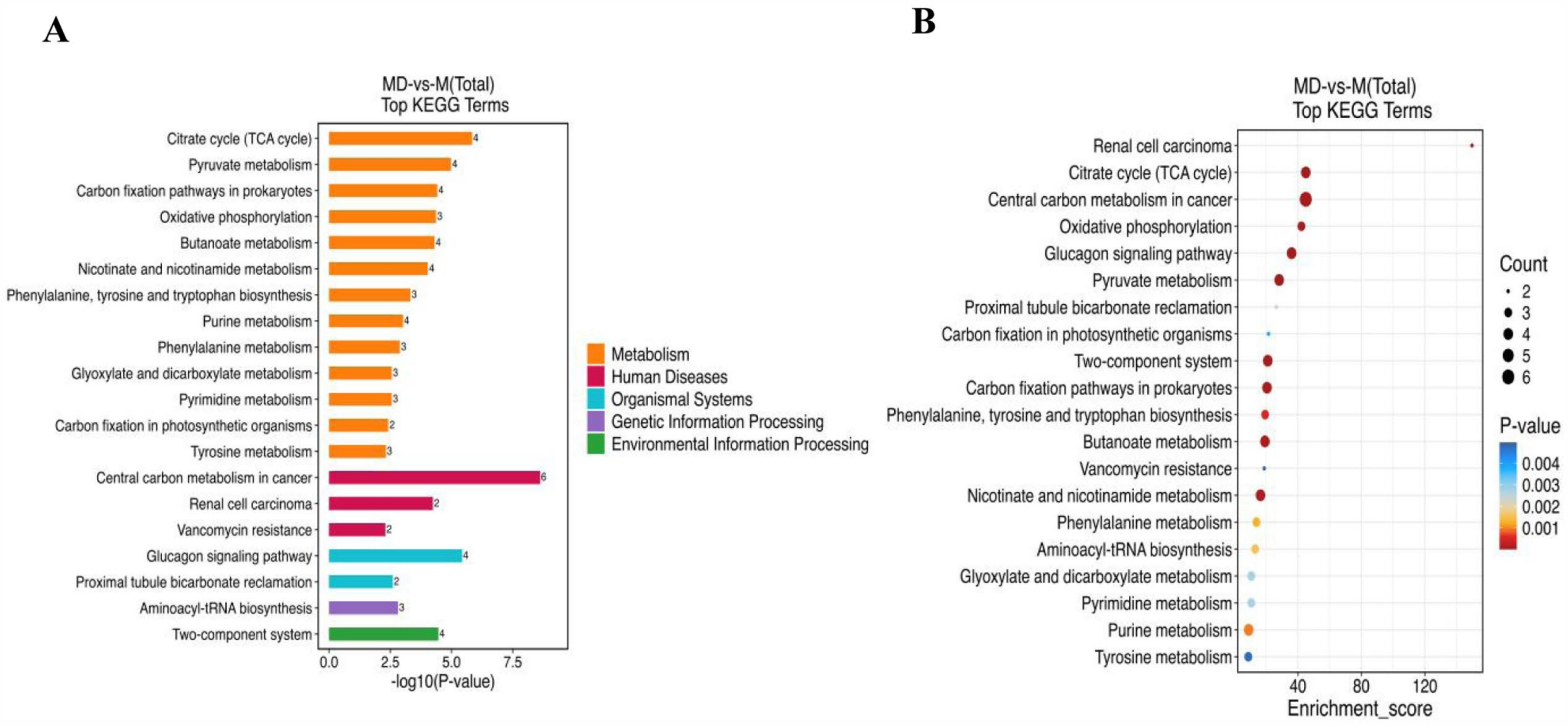
The top 20 pathways of significate of the upregulated and down-regulated differential metabolites on KEGG. **(A)** Differential metabolites quantity in enrichment pathway; **(B)** KEGG bubble Chart (Top 20).

**Figure 10:**
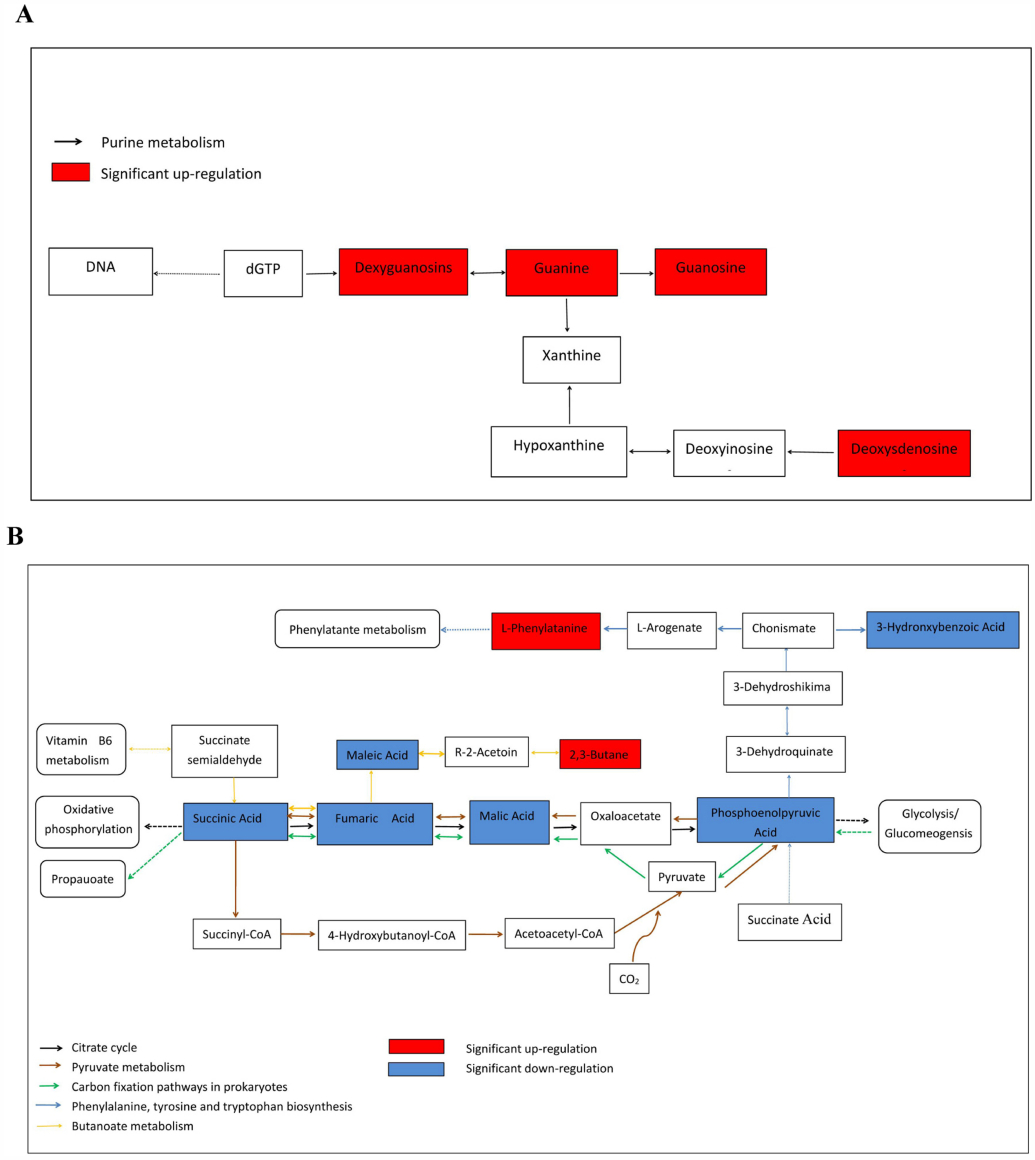
Abundance of the metabolites enriched in metabolism compared intercropping treatment with monoculture treatment. **(A)** Purine metabolism; **(B)** citrate cycle,Pyruvate metabolism, Carbon fixation pathways in prokaryotes, Phenyalanine tyrosine and tryptophan biosynthesis and butanoate metabolism. The figure only shows some metabolic pathways involved in the results of this experiment.

Based on the Kyoto Encyclopedia of Genes and Genomes database, metabolic pathway enrichment analysis was performed for the differential metabolites of the M and MD models. Among the top 20 pathways with the largest number of enriched metabolites, 65.67% belonged to the metabolic pathways (Figure 9A). The top 20 pathways with the most significant enrichment were the citrate cycle (TCA cycle), pyruvate metabolism, carbon fixation pathways in prokaryotes, phenylalanine, tyrosine and tryptophan biosynthesis, butanoate metabolism, and urine metabolism (Figure 9B). These pathways were involved in the anabolism of carbohydrates, alcohols, purine nucleotides, amino acids, dicarboxylic acids, hydroxyl acids, and their derivatives, and 12 metabolites with significant differences were involved in these 6 metabolic pathways. Among them, 2,3-butanediol, L-phenylalanine, deoxyguanosine, guanosine, guanine, and deoxyadenosine levels increased significantly in the MD mode. Fumaric acid, succinic acid, phosphoenolpyruvic acid, malic acid, maleic acid, and 3-hydroxybenzoic acid level decreased significantly in the MD mode. These 12 metabolites were exhibited a strong association (Figures 10A,B). Therefore, in cassava soil, the cassava–soybean intercropping pattern affects the citrate cycle (TCA cycle), pyruvate metabolism, carbon fixation pathways in prokaryotes, phenylalanine, tyrosine and tryptophan biosynthesis, butanoate metabolism, purine metabolism, and other metabolic pathways. It promoted the accumulation of alcohol, purine nucleotides and amino acids such as 2,3-butanediol, L-phenylalanine, deoxyguanosine, guanosine and deoxyadenosine and inhibited the accumulation of fumaric acid, succinic acid, phosphoenolpyruvic acid, malic acid, maleic acid, 3-hydroxybenzoic acid, and other organic acids.

### 3.5 Correlation analysis of soil physicochemical properties, different microorganisms, and different metabolism

**Figure 11:**
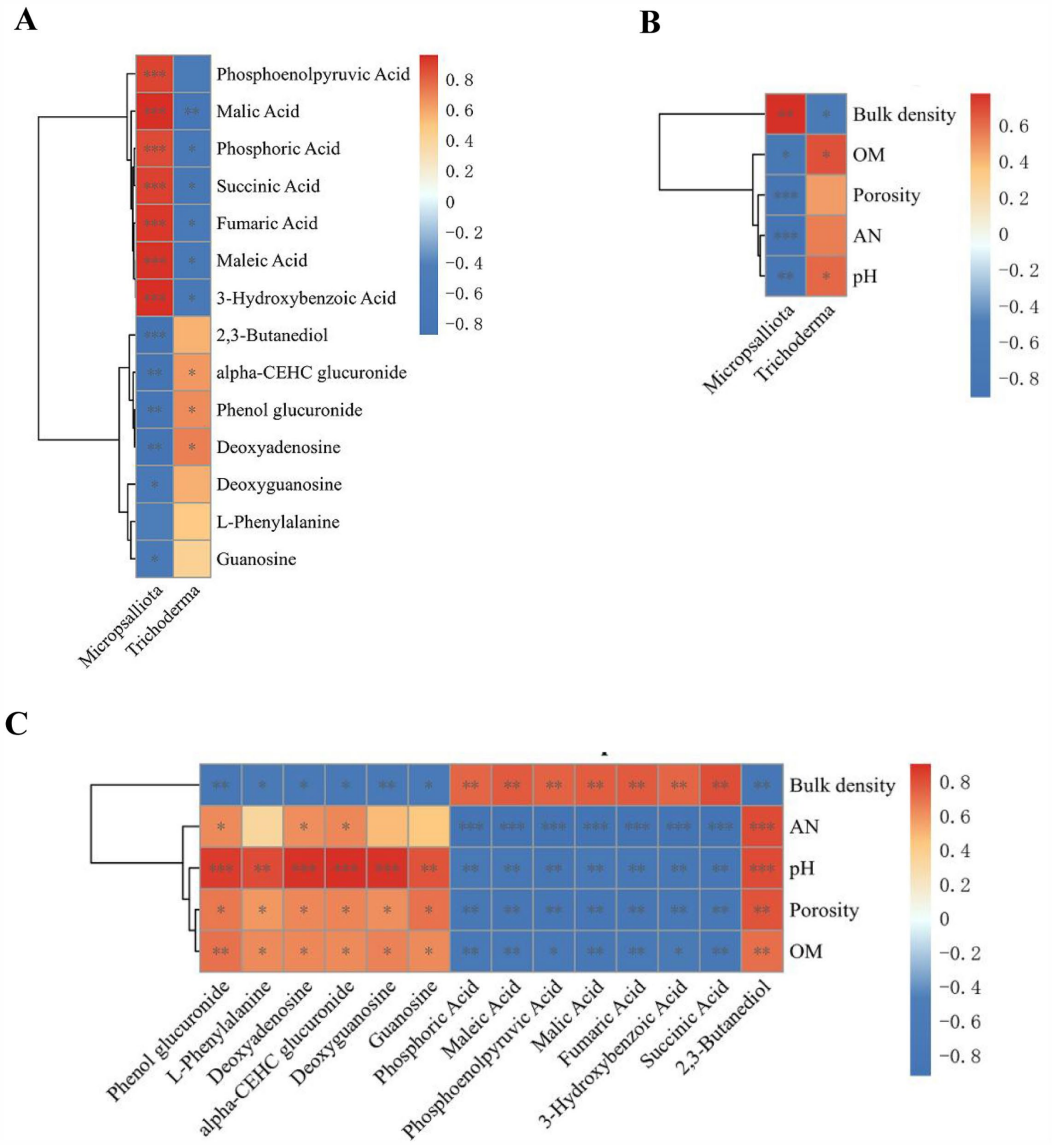
Spearman’s correlations of significant differential metabolites, fungal biomarkers (LDA score > 3.5) and soil physicochemical properties (*p* < 0.05). **(A)** Metabolites vs. fungal biomarkers correlation; **(B)** soil physicochemical properties vs. fungal biomarkers correlation; and **(C)** metabolites vs. soil physicochemical properties correlation. Significance between metabolites, fungal biomarkers and physicochemical properties: *, *p* ≤ 0.05, **, *p* ≤ 0.01, ***, *p* ≤ 0.001.

Spearman’s correlation analysis was conducted between the screened significantly different metabolites, fungal markers, and soil physicochemical properties, and the correlations are presented as matrix heat maps (Figures 11A–C). The clustering results showed that *Trichoderma* was significantly negatively correlated with organic acids, such as fumaric acid, succinic acid, phosphoenolpyruvic acid, malic acid, maleic acid, 3-hydroxybenzoic acid, and soil bulk density. However, there was a significant positive correlation between pH and carbohydrates, nucleotide substances, and organic acids, such as phenol glucuronide and alpha-CEHC deoxyguanosine. Fumaric acid, succinic acid, phosphoenolpyruvic acid, malic acid, maleic acid, and 3-Hydroxybenzoic acid were significantly positive correlated with soil bulk density but negatively correlated with organic acid, AN, soil porosity, and pH. Carbohydrates, alcohols, and purine nucleotides such as phenolglucuronide, Alpha-CEHC glucuronide, 2,3-butanediol, L-phenylalanine, deoxyguanosine, guanosine, and deoxyadenosine were significantly positively correlated with glycosides, amino acids, organic acid, AN, soil porosity, and pH. Based on the relationship between the significantly different metabolites, fungal markers, and soil physicochemical properties, it can be seen that the cassava–soybean mode can promote the accumulation of carbohydrates, alcohols, purine nucleotides, and amino acids such as phenolglucuronide, 2,3-butanediol, L-phenylalanine and deoxyguanosine in soil and inhibit the excessive accumulation of organic acids such as fumaric acid, succinic acid, and phosphoenolpyruvic acid. In addition, the soybean cassava intercropping mode recruited the beneficial fungal genus *Trichoderma*, which interacted with soil metabolites to promote the increase in soil porosity, OM, AN, and pH. This improved the adverse factors of severe soil acidification, soil compaction, and nutrient decline in continuous cassava cropping and alleviated its limitations.

## 4 Discussion

Cassava has serious continuous cropping limitations, and the current main method to alleviate these limitations is to change fertilisation strategies (Wang, 2016; Wei et al., 2017). However, this method requires more economic investment, and improper fertilisation may further exacerbate limitations to continuous cropping obstacles. Therefore, it is crucial to develop an efficient and eco-friendly method to alleviate continuous cassava cropping obstacles. This study explored the effect of soybean intercropping on alleviating continuous cropping obstacles in cassava, which is the first attempt to apply intercropping to alleviate these obstacles.

Intercropping is considered a sustainable ecological agricultural planting model that improves land use efficiency and crop yield, while also altering the microclimate conditions of farmland (Agegnehu et al., 2008; Cong et al., 2015), thereby improving soil nutrient and pH values (Liu et al., 2014; Li et al., 2018). Reasonable interplanting of legumes can effectively improve soil physicochemical properties and fertility (Wang et al., 2014; Solanki et al., 2019). For example, the zonal intercropping model of corn–soybean could increase soil OM and nitrogen content (Te et al., 2022) and reduce soil bulk density (Peng et al., 2024). Interplanting quinoa–red beans significantly increases soil AN, OM, and other nutrients (Xue et al., 2023). Oil tea–soybean interplanting can improve soil physicochemical properties and soil pH and effectively increase soil OM, nitrogen, phosphorus, and potassium contents (Li et al., 2013). Soybean–tea interplanting increases the soil ammonium nitrogen content (Duan et al., 2019). *Rhizobium* legumes have a nitrogen-fixing effect, which can convert nitrogen in the air into nitrogen-containing substances that can be absorbed by plants and effectively improve the AN content in the soil (Bo et al., 2018). In addition, during the growth of soybeans in acidic soil, soybean roots can promote organic nitrogen mineralisation and release more OH-; however, they can inhibit the organic acid secretion of other plant roots (Hua et al., 2018), which improves the soil pH to a certain extent. Changes in tillage methods can lead to changes in soil physical and chemical properties and microorganisms, which directly affect the decomposition and transformation of soil OM and ultimately affect its content (Segura et al., 2019; Liu M. et al., 2020). The effect of cassava–soybean intercropping on the physicochemical soil properties of continuous cassava cropping is mainly attributed to nitrogen-fixing bacteria and exudates of leguminous roots.

Cassava–soybean intercropping can significantly increase the number of fungi and bacteria in continuous cassava cropping soil and has a greater impact on the community composition and relative abundance of dominant fungi, among which the abundance of the beneficial fungus *Trichoderma* was most significant. This indicated that cassava–soybean intercropping changed the microbial structure of the continuous cassava cropping soil and recruited a large number of beneficial *Trichoderma* fungi, improving the soil microecology of continuous cassava cropping. The soil microbial structure changes substantially in many soybean interplanting systems. For example, the number of operational taxonomic units (OTUs) of bacteria and fungi in the sugarcane–soybean intercropping system is higher than that in the monoculture sugarcane model (Malviya et al., 2021), and the abundance of important fungal genera, such as *Trichoderma, Sarcoidales*, and *Fusarium,* increased (Lian et al., 2018). Maize–soybean interplanting changes the diversity and composition of the soybean rhizosphere pathogen *Fusarium* and biocontrol fungal communities, which significantly inhibits the occurrence of soybean root rot (Chang et al., 2022). Intercropping of wheat and broad beans results in a change in the microbial community structure of the rhizosphere soil and a significant decrease in the number of sickles (Guo, 2022). The soybean interplanting system can change the structure of soil microorganisms in continuous cassava cropping, which may be because the intermediate planting of soybeans in this study increased the soil pH value and soil nitrogen and OM content, providing a substrate for microbial growth or improving the microenvironment of the microbial habitat (Lian et al., 2018; Ameloot et al., 2021), ultimately contributing to an increase in soil microbial abundance. Interestingly, the interplanting of different crops and legumes had different effects on soil microorganisms. This may be because the root exudates of different crops are different, resulting in different coupling mechanisms among root exudates, microorganisms, and soil physicochemical properties, which also highlights the importance of studying microecological diversity in different soybean interplanting systems.

Soil metabolites are derived from plant root exudates, microbial metabolites, and the decomposition of plants, microorganisms, and soil organic matter and play an important role in plant growth (Cheng et al., 2018; Kuzyakov and Razavi, 2019). Intercropping systems change soil microorganisms, as well have a complex effect on the accumulation of soil metabolites. Wheat–broad bean intercropping significantly enriches soil flavonoids (Liu Y. C. et al., 2020). Maize–peanut and potato–maize intercropping systems alter the type and content of root exudates (Li et al., 2020; Liu K. et al., 2020). We found that cassava–soybean intercropping promoted the accumulation of carbohydrates, alcohols, purine nucleotides, and amino acid metabolites, such as phenolglucuronide, 2.3-butanediol, L-phenylalanine, and deoxyguanosine. Further, it inhibited the accumulation of fumaric acid, succinic acid, phosphoenolpyruvic acid, malic acid, maleic acid, 3-Hydroxybenzoic acid, and other organic acids. In some interlegume systems, changes in metabolites, such as carbohydrates, alcohols, amino acids, purine nucleotides, and organic acids, are observed. For example, adenine, adenosine, and maltose contents in the rhizosphere soil of a sugarcane–peanut intercropping system increased significantly (Tang et al., 2020). Alcohol metabolites are markedly enriched in tea tree–small bean intercropping (Wang et al., 2023). Maize–soybean intercropping significantly enriches amino acid metabolites, such as indole-3-carboxyaldehyde (OA02), 1H-indole-3-carboxylic acid (OA03, OA04), erucic acid (OA06), and L-glutamic acid (AA01), in maize rhizosphere soil (Xu et al., 2023). Wheat–broad bean intercropping can increase the content of tartaric acid, malic acid, and other organic acids in the rhizosphere soil (Lv et al., 2021). Because soil metabolites are root-microbial co-produced compounds, we cannot determine the main source of differential metabolites; however, it can be inferred that increased carbohydrates, alcohol metabolism, amino acids, and purine nucleotides in soybean intercropping systems are closely related to the symbiosis of soybean and root nodules. The carbohydrates produced by legumes through photosynthesis supply the growth and development of soybeans, while also serving as a source of nitrogen-fixing bacteria through root exudates (Ke et al., 2022), and purine nucleosides, such as inosine phosphate (IMP), play a key role in the nitrogen fixation of soybean root nodules (Claudio et al., 2023; Luisa et al., 2022), and some amino acids and alcohols are important signal substances in the soy-root nodule symbiosis process (Lodwig et al., 2003). Therefore, the effect of the cassava–soybean intercropping system on the accumulation of metabolites in cassava soil is mainly due to the symbiotic relationship between soybeans and nitrogen-fixing bacteria.

Soil microorganisms and their metabolites play important roles as soil nutrients. In this experiment, phenolglucuronide, 2.3-butanediol, L-phenylalanine, deoxyadenosine, and other carbohydrates, alcohols, purine nucleotides, and amino acids were significantly positively correlated with soil OM and AN. *Trichoderma* fungi showed a significant positive correlation with soil OM, and these results were similar to those of previous studies (Bais et al., 2006; Holz et al., 2018; Zhu et al., 2021). Carbohydrates, alcohols, purine nucleotides, and amino acids are OM components. Amino acids can be absorbed by microorganisms and release large amounts of ammonium nitrogen in the form of NH_4_^+^ (Ma et al., 2021). *Trichoderma* can accelerate the degradation of OM in the soil, such as lignin in straw (Kamimur et al., 2019; German et al., 2015), which is the main reason why carbohydrates, alcohols, purine nucleotides, amino acids, and *Trichoderma* fungi are closely related to soil OM and AN. Currently, there are few studies on the relationship between soil microorganisms and metabolites and soil porosity and bulk density, and it remains largely unclear. However, the *Trichoderma* fungi, carbohydrates, alcohols, purine nucleotides, and amino acids in this study were significantly positively correlated with soil porosity, which is worthy of further exploration. In addition, this study also found that fumaric acid, succinic acid, phosphoenolpyruvic acid, and other organic acids were significantly negatively correlated with pH. They significantly decreased in the cassava–soybean intercropping system, indicating that the main cause of soil acidification in continuous cassava cropping was the excessive accumulation of organic acids. The accumulation of organic acids in the soil was effectively curbed by intercropping with soybeans, thereby alleviating soil acidification. The interaction between soil microorganisms and metabolites is a complex and important network that jointly affects soil ecology, plant growth, and development (Cheng et al., 2022; Su et al., 2023). For example, *Trichoderma* fungi can promote the degradation of carbohydrates such as plant residues. After the degradation of plant residues, large amounts of carbohydrates and nucleotides are released into the soil, and the decomposed carbohydrates provide sufficient carbon and energy for the reproduction of *Trichoderma* fungi (Mendoza-Mendoza et al., 2018). This indicated that *Trichoderma* fungi had a positive correlation with soil metabolites, carbohydrate, and nucleotides. Therefore, the interaction between soil metabolites and microorganisms under cassava–soybean intercropping jointly affected the physical and chemical properties of cassava soil, thereby promoting the growth of cassava and alleviating the obstacles of cassava continuous cropping.

## 5 Conclusion

In continuous cassava cropping soil, the intercropping of cassava–soybean can promote the accumulation of carbohydrates, alcohols, purine nucleotides, and amino acids, such as 2,3-butanediol, L-phenyalanine, deoxyguanosine, guanosine, and deoxyadenosine; inhibit the excessive accumulation of organic acids such as fumaric acid, succinic acid, phosphoenolpyruvic acid, malic acid, maleic acid, and 3-Hydroxybenzoic acid; and recruit beneficial fungal genus *Trichoderma. Trichoderma* interacts with soil metabolites to increase soil porosity, pH, organic matter and available nitrogen content, thereby improving the adverse factors of severe soil acidification, soil compaction, and nutrient decline in continuous cassava cropping, effectively alleviating the obstacles of continuous cassava cropping.

## Data Availability

The datasets presented in this study can be found in online repositories. The names of the repository/repositories and accession number(s) can be found at: https://www.ncbi.nlm.nih.gov/, PRJNA1184020.
